# Axon Injury-Induced Autophagy Activation Is Impaired in a *C. elegans* Model of Tauopathy

**DOI:** 10.3390/ijms21228559

**Published:** 2020-11-13

**Authors:** Su-Hyuk Ko, Gilberto Gonzalez, Zhijie Liu, Lizhen Chen

**Affiliations:** 1 Barshop Institute for Longevity and Aging Studies, University of Texas Health Science Center at San Antonio, San Antonio, TX 78229, USA; KOSH@uthscsa.edu; 2 Department of Cell Systems and Anatomy, University of Texas Health Science Center at San Antonio, San Antonio, TX 78229, USA; gonzalezg11@livemail.uthscsa.edu; 3 Department of Molecular Medicine, University of Texas Health Science Center at San Antonio, San Antonio, TX 78229, USA; liuz7@uthscsa.edu

**Keywords:** tauopathy, autophagy, aggregation, Tau, ptl-1, axon injury, axon regeneration

## Abstract

Autophagy is a conserved pathway that plays a key role in cell homeostasis in normal settings, as well as abnormal and stress conditions. Autophagy dysfunction is found in various neurodegenerative diseases, although it remains unclear whether autophagy impairment is a contributor or consequence of neurodegeneration. Axonal injury is an acute neuronal stress that triggers autophagic responses in an age-dependent manner. In this study, we investigate the injury-triggered autophagy response in a *C. elegans* model of tauopathy. We found that transgenic expression of pro-aggregant Tau, but not the anti-aggregant Tau, abolished axon injury-induced autophagy activation, resulting in a reduced axon regeneration capacity. Furthermore, axonal trafficking of autophagic vesicles were significantly reduced in the animals expressing pro-aggregant F3ΔK280 Tau, indicating that Tau aggregation impairs autophagy regulation. Importantly, the reduced number of total or trafficking autophagic vesicles in the tauopathy model was not restored by the autophagy activator rapamycin. Loss of PTL-1, the sole Tau homologue in *C. elegans*, also led to impaired injury-induced autophagy activation, but with an increased basal level of autophagic vesicles. Therefore, we have demonstrated that Tau aggregation as well as Tau depletion both lead to disruption of injury-induced autophagy responses, suggesting that aberrant protein aggregation or microtubule dysfunction can modulate autophagy regulation in neurons after injury.

## 1. Introduction

Autophagy is a lysosome-mediated intracellular catabolic process that is a central component in stress response [1]. It plays a conserved role in maintaining cellular homeostasis by degrading dysfunctional proteins, lipids, and organelles through an autophagosome-lysosome pathway [2]. In response to increased energy demands of cells, autophagy provides metabolic substrates, making autophagy a cytoprotective mechanism [3]. Autophagy was first discovered in conditions of starvation, which activates autophagy [4]. Autophagy is initiated with the formation of a double membrane structure around cellular substances, followed by the formation of autophagosome that then fuses with a lysosome. The engulfed substances are then degraded and recycled back to the cell as amino and fatty acids [5].

Connections between autophagy and human disease or physiology have been active research topics. It has become apparent that autophagy plays a central role not only in cancer and aging but also in neurodegeneration. As neurons become terminally differentiated post-mitotic cells early in development, they are unable to dilute out misfolded proteins and damaged organelles through cell division like replicating cells [6]. The mice lacking the essential autophagy-related geneAtg7 in the central nervous system showed massive neuronal degenerations in the cerebral and cerebellar cortices [7]. Ineffective autophagic lysosomal clearance, resulting in toxic protein accumulation, is found in various neurodegenerative diseases [3]. Tauopathies in particular share a commonality in their aggregation of either wild-type or mutant, phosphorylated Tau. A key role of autophagy in tauopathies is the removal of aggregated Tau [8,9,10]. Autophagy activators have been shown to reduce the levels of misfolded and aggregated proteins, mitigating the spreading of tau and neuronal loss [11,12,13,14,15,16,17], although whether autophagy impairment is a contributor or a consequence of tauopathy remains unclear [18,19].

Accumulating evidence also supports that proper function of autophagy is critical for the maintenance of normal axonal function by supporting local axon homeostasis and protecting against axonal degeneration under stress conditions [20,21,22,23]. An explicit acute neuronal stressor is axonal injury with extreme responses involving calcium influx, axonal membrane sealing, injury signaling and transcriptional changes [24]. Previous studies in rodent models reveal that autophagy is among the cellular process altered after axon injury [25,26,27]. Axon injury has been shown to up-regulate essential autophagy genes, including *Ambra*, *Atg5*, *Beclin 1* and *LC3* in both CNS and PNS [28,29]. Activating autophagy after spinal cord injury lesion attenuated axonal retraction and promotes axon regeneration by stabilizing microtubules [28]. Using a reporter that monitors autophagosomes and autolysosomes, we have recently reported that axon injury triggers autophagy activation, which declines with age, and that the injury-induced autophagy activation is critical for axon regeneration by limiting NOTCH [30]. However, the injury-induced autophagy response has not been previously investigated in the context of neurodegeneration.

In this study, we monitor the dynamics of autophagosomes and autolysosomes in response to axon injury in a *C. elegans* model of tauopathy that expresses pro-aggregant F3ΔK280 Tau fragment [31]. We found that pro-aggregant Tau abolished axon injury-triggered autophagy activation and reduced axon regeneration capacity. In addition, axonal trafficking of autophagic vesicles were significantly reduced in the tauopathy model. The autophagy activator rapamycin failed to rescue the defects in autophagy activation or trafficking caused by Tau aggregation. Loss of PTL-1, the sole Tau homologue in *C. elegans*, also led to impaired injury-induced autophagy activation, with an increased basal level of autophagic vesicles. Therefore, like in aged neurons, injury-triggered autophagy induction is impaired in neurons with Tau aggregation or Tau depletion, suggesting a negative impact of aberrant protein aggregation and MT dysfunction on neuronal autophagy response to injury.

## 2. Results

### 2.1. Axon Injury-Triggered Autophagy Induction Is Abolished in a Transgenic Tauopathy Model

**Figure 1:**
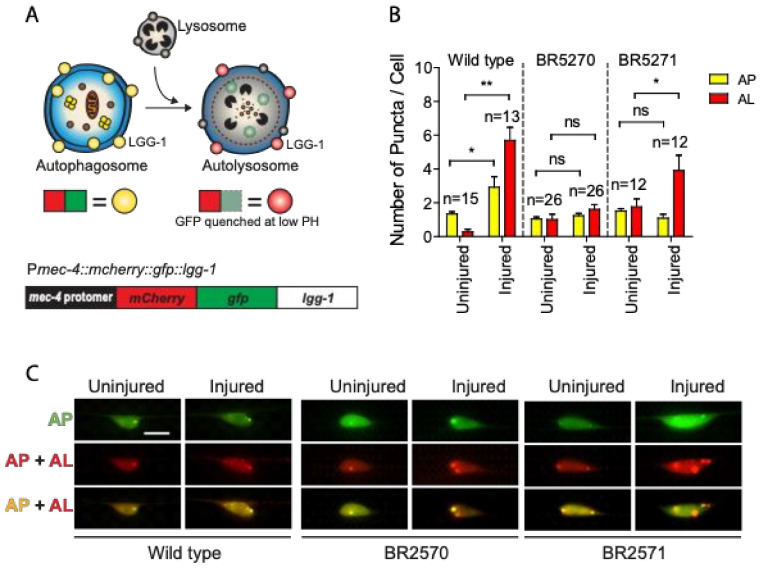
Axon injury-induced autophagic induction is impaired in transgenic Tauopathy models. (**A**) Schematic diagram showing the mCherry::GFP::LGG-1 reporter specifically expressed in PLM touch neuron. (**B**,**C**) Quantification and representative images of PLM cell body of wild type, BR5270 and BR5271 animals expressing P*mec-4*-mCherry::GFP::LGG-1. PLM axon was axotomized on Day 1 of adulthood. Images were taken immediately before axotomy (Uninjured) and 24 h post axotomy (injured). AP, autophagosome; AL, autolysosome. Scale bar: 10 µm. Statistics: one-way ANOVA; mean ± SEM; * *p* < 0.05; ** *p* < 0.01; ns, Not significant.

Previous studies have suggested that autophagy dysfunction contributes to the toxic aggregation in neurodegenerative diseases. Conversely, increasing evidence also implicates the protein aggregation itself in affecting autophagy regulation [32]. We have recently reported that axon injury activates autophagy, which is required for effective axon regeneration through limiting Notch signaling [30]. To test whether injury-induced autophagy is affected by protein aggregation, we investigated autophagy dynamics in a transgenic Tau aggregation model that expresses chromosomally integrated versions of the amyloidogenic F3DK280 fragment of human Tau derived from the repeat domain of TauDK280 [31,33,34]. We expressed a dual-fluorescent mCherry::GFP::LGG-1 protein under the control of a touch neuron-specific promoter (Figure 1A) in the strains carrying integrated transgene of the pro-aggregant F3ΔK280 (BR5270 *byIs161*) or the anti-aggregant F3ΔK280-PP control (BR5271 *byIs162*). The dual-fluorescent reporter has been previously used to monitor both autophagosomes and autolysosomes, and has been shown to be functional as it was able to rescue an embryonic lethal *lgg-1(tm3489)* mutant [35,36]. In cells expressing this reporter, autophagosomes are positive for both GFP and mCherry, while autolysosomes are only positive for mCherry, as GFP is quenched due to the low pH in autolysosomes.

As we previously reported [30], we observed significantly increased numbers of mCherry/GFP double-positive autophagosome puncta and mCherry single-positive autolysosome puncta in injured PLM neurons compared to uninjured neurons in wild-type animals (Figure 1B,C), indicating that axon injury induces autophagic vesicle formation and promotes autophagy flux. This injury-induced autophagy activation was completely abolished in the BR5270 strain expressing pro-aggregant Tau, although we were able to detect a significantly enhanced number of autolysosomes puncta in the control strain BR5271 (Figure 1B,C). These aforementioned findings suggest that Tau aggregation impairs the autophagy response to axon injury.

### 2.2. Transgenic Expression of Pro-Aggregant Tau Leads to Reduced Axon Regeneration after Injury

**Figure 2:**
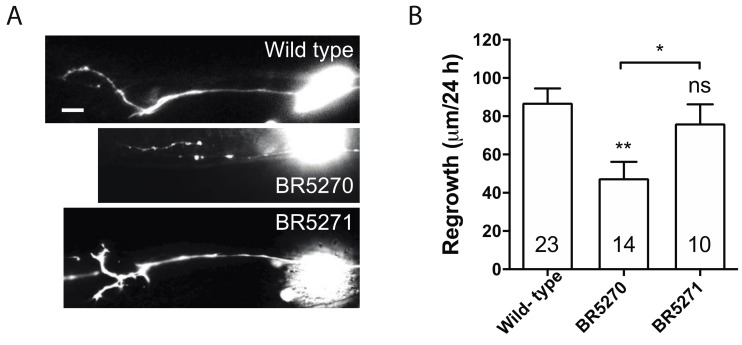
Pro-aggregant Tau impairs axon regeneration post injury. (**A**) Representative images of PLM axon regeneration 24 h post-laser axotomy in the wild-type animals, BR5270 strain expressing pro-aggregant Tau, and BR5271 strain expressing anti-aggregant Tau. PLM axon is visualized by touch neuron-specific GFP expression driven by a *mec-7* promoter. Scale bar: 10 µm. (**B**) Quantification of axon regeneration for experiments in (**A**). Statistics: one-way ANOVA; mean ± SEM; * *p* < 0.05; ** *p* < 0.01; ns, Not significant.

Our previous studies implicated only autophagic activation in response to injury, as opposed to basal autophagy itself, is correlated with the regeneration capacity of axons [30]. In addition, this response is reduced with age [30]. This age-related diminishing of autophagic induction correlates to the gradually reduced axon regeneration in PLM neurons [37]. In *dlk-1* mutant, in which PLM axon regrowth is completely inhibited, axotomy fails to induce autophagy, although loss of DLK-1 does not affect the basal autophagy level in non-injured neurons [30]. Similarly, blocking internal calcium release is sufficient to inhibit axon regrowth [38] and block autophagy induction after injury [30]. As transgenic expression of pro-aggregant Tau abolished injury-triggered autophagy response, we hypothesized that it would also diminish axon regeneration. Indeed, PLM axon regrowth in BR5270 was significantly less than that in wild-type control, while regrowth in BR5271 was comparable to wild-type (Figure 2A,B). This supports our claim that injury-induced autophagy activation correlates with axon regeneration capacity.

### 2.3. Injury Induces Autophagic Vesicles Trafficking in Wild-Type Neurons but Not in Neurons Expressing Pro-Aggregant Tau

**Figure 3:**
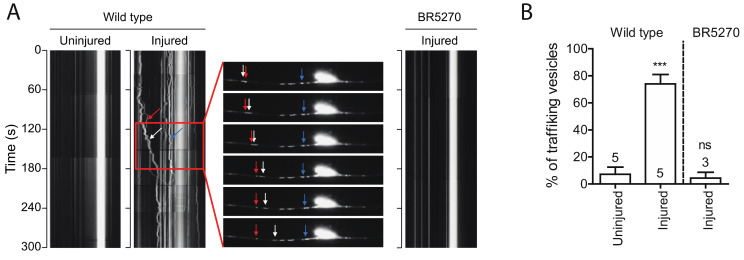
Injury induces autophagic vesicles trafficking in wild-type animals but not in tauopathy models. (**A**) Representative kymograph of autophagic vesicles movement in touch neuron (visualized with P*mec-4*-mCherry::GFP::LGG-1 reporter); each arrow (red, blue, and white) indicate autophagic vesicles on PLM axon. Kymographs were generated by Olympus IX83 software from movies taken immediately before axotomy (Uninjured) and 24 h post axotomy (injured). (**B**) Quantification of trafficking % (number of trafficking vesicles/total vesicles in 50 µm region of proximal PLM axon) for experiments in (**A**). Statistics: one-way ANOVA; mean ± SEM; *** *p* < 0.001; ns, Not significant.

Autophagic vesicles have been shown to move bidirectionally in axons of primary neurons [39]. We observed very few moving autophagic vesicles labeled by GFP::LGG-1 in intact PLM axons in wild-type young adult animals. Remarkably, the portion of moving vesicles significantly increased in regenerating axons 24 h post injury, with majority of the puncta moving retrogradely (Figure 3A,B). This observation is consistent with previous studies showing that autophagosomes move bidirectionally along MTs and ultimately concentrate around the centrosome in the perinuclear region MT minus-end-directed motor dynein [40]. However, when we examined the vesicle trafficking in BR5270 strain, we did not detect this injury-associated movement of autophagic puncta (Figure 3A,B), possibly due to the inhibited autophagosome formation by pro-aggregant Tau.

### 2.4. Autophagy Activator Rapamycin Failed to Rescue Autophagy Defects in the Tauopathy Model

**Figure 4:**
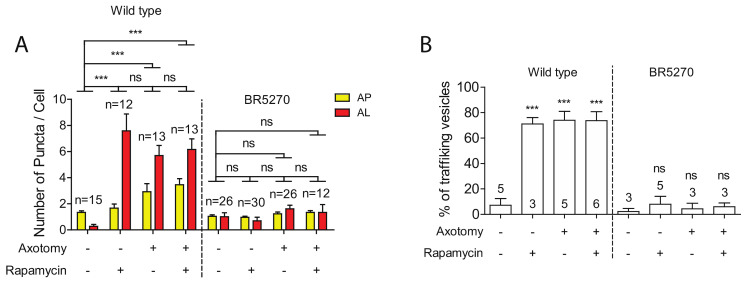
Defects in autophagic induction and trafficking are not rescued by Rapamycin. (**A**) Quantification of autophagosomes (APs) and autolysosomes (ALs) in PLM cell bodies with indicated treatments. Rapamycin or axotomy alone was sufficient to enhance the numbers of APs and ALs in PLM neurons of wild type. In contrast, neither rapamycin nor axon injury was able to induce the formation of autophagic vesicles in PLM neurons of the Tauopathy model. (**B**) Quantification of autophagic vesicles movement in the PLM axons with indicated treatments. Rapamycin or axotomy alone was sufficient to enhance trafficking in wildtype animals, but not in BR5270. Statistics: one-way ANOVA; mean ± SEM; *** *p* < 0.001; ns, Not significant.

Autophagy activators, such as rapamycin, have been shown to reduce the levels of aggregated Tau proteins, mitigating the Tau aggregation-induced neurodegeneration phenotypes [11,12,13,14,15,16,17]. Given this, we explored whether rapamycin was able to rescue the defects in both autophagy induction and trafficking in neurons expressing pro-aggregant Tau. As we have previously reported [30], rapamycin treatment was able to significantly enhance the numbers of autolysosome puncta in intact PLM neurons, although it was not sufficient to further increase autophagic vesicles in injured neurons (Figure 4A). This is possibly due to the already high basal level of autophagy in injured young neurons, as rapamycin treatment was sufficient to elevate the number of autophagic vesicles in injured old neurons, which had shown diminished autophagic response to injury [30]. Like aged neurons, neurons expressing pro-aggregant Tau failed to activate autophagy in response to axon injury (Figure 1 and Figure 4A). We therefore predicted that rapamycin would at least partially restore the autophagic response to injury in the tauopathy model. However, rapamycin treatment was found to not increase autophagic vesicles in the presence of pro-aggregant Tau, despite the low basal level.

We next examined the effect of rapamycin on autophagic vesicle trafficking. Rapamycin treatment greatly enhanced the percentage of moving autophagic vesicles in uninjured PLM axons of wild-type animals. Either rapamycin or axotomy alone was sufficient to promote the trafficking of autophagic vesicles, whereas combining both treatments did not further enhance trafficking (Figure 4B). We also examined rapamycin effect on trafficking in BR5270 strain. No change was observed treated and untreated neurons in both intact and injured neurons (Figure 4B), consistent with the inability of rapamycin in promoting autophagic vesicle formation.

### 2.5. Loss of PTL-1 Blocks Injury-Induced Autophagy Activation

**Figure 5:**
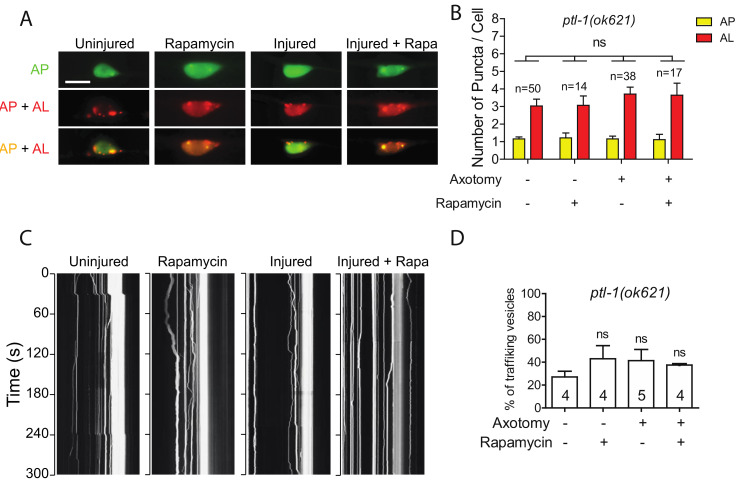
Defective autophagic induction and trafficking in *ptl-1* mutant. (**A**,**B**) Representative images and quantification of APs and ALs in PLM cell bodies of *ptl-1(ok621)* expressing P*mec-4*-mCherry::GFP::LGG-1 with indicating treatment. Rapamycin treatment did not rescue the autophagic activity in PLM neurons of the *ptl-1* mutant animals. Scale bar: 10 µm. (**C**) Representative kymograph of autophagic vesicles movement in touch neuron with indicating treatment. Axotomy or axotomy with rapamycin treatment did not enhance the movement of the autophagic vesicles on the PLM axon of *ptl-1(ok621)*. (**D**) Quantification of autophagic vesicles movement in the PLM axons with indicated treatments (measured as number of trafficking vesicles/total vesicles on PLM axon). Statistics: one-way ANOVA; mean ± SEM; ns, Not significant.

Like many other membrane-bound organelles and vesicles, autophagosome dynamics rely in part on their interactions with the cytoskeleton and especially with microtubules (MTs) [41]. Tau is a MT-binding protein that promotes MT assembly and stability. In Tau-induced neurodegeneration, Tau aggregation is often associated with Tau hyperphosphorylation and loss of Tau-MT interaction [42]. These prompted us to examine how Tau depletion affected injury-triggered autophagy induction using a genetic mutant allele of *ptl-1* gene, which encodes the sole ortholog of human MAPT in *C. elegans* [43,44]. Interestingly, we observed a higher number of autolysosomes in uninjured PLM neurons of *ptl-1(ok621)* mutants (Figure 5A,B). It has been proposed that MTs are not involved in autophagosome formation under basal conditions, as several studies have reported that acute treatment of a MT destabilization drug, nocodazole, as well as a MT stabilization drug, Taxol, did not affect basal autophagosome formation [45,46,47]. The high basal autophagy level of autophagy in *ptl-1* mutant might be due to prolonged loss of function of PTL-1 in the mutant, possibly resulting in a stress-like condition. We found that both axotomy and rapamycin treatment failed to promote autophagic vesicle formation in *ptl-1* mutant (Figure 5A,B), thus indicating the important role of MTs in autophagy regulation.

We also observed higher basal level of autophagic vesicle trafficking in intact PLM axons in *ptl-1* mutants (Figure 5C,D). Unlike in the wild-type animals, rapamycin treatment did not significantly enhance trafficking in intact axons in *ptl-1* mutants. Axotomy also failed to promote trafficking in the absence of PTL-1 (Figure 5C,D). Therefore, these findings suggest that loss of PTL-1 leads to diminished autophagic response to axon injury, despite the enhanced basal level of autophagy.

### 2.6. The High Basal Level of Autolysosomes in Ptl-1 Mutant Is Resistant to Autophagy Inhibitor BA1

**Figure 6:**
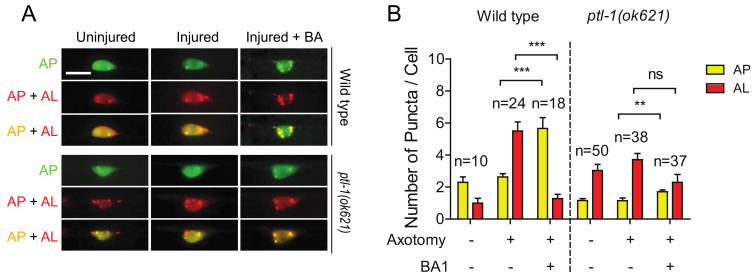
The high basal level of autolysosomes in the *ptl-1* mutant is less sensitive to BA1 compared to wild type. (**A**) Representative images of both wild-type and *pt1-1(ok621)* transgenic PLM cell bodies, expressing P*mec-4*-mCherry::GFP::LGG-1, 24 h post axotomy. Images taken with and without injection of 50µM of BA1 into tail region. Treatment occurred at day 1 of adulthood 1 h before axotomy. BA1 suppressed autolysosome formation in injured wild-type neurons, but not in *ptl-1(ok621)* mutants. Scale bar: 10 µm. (**B**) Quantification of APs and ALs in PLM cell bodies for experiments in (**A**). Statistics: one-way ANOVA; mean ± SEM; ** *p* < 0.01; *** *p* < 0.001; ns, Not significant.

Bafilomycin A1 (BA1) is a wildly used inhibitor of the late phase autophagy. BA1 blocks vacuolar type H^+^-ATPases and prevents maturation of autophagic vacuoles by inhibiting fusion between autophagosomes and lysosomes. As we previously reported [30], BA1 treatment by injection into the body cavity resulted in an increase in mCherry/GFP double positive puncta (indicating an increase of autophagosomes) and a decrease in mCherry single positive puncta (indicating a decrease of autolysosomes) in injured neurons of wild-type animals (Figure 6A,B). Ample evidence from previous studies has shown that MTs are involved in the formation and motility of autophagosomes, but not in the process of autophagosome fusion with lysosomes [48]. We found that BA1 treatment did not significantly affect the numbers of autolysosomes in injured neurons in *ptl-1* mutant (Figure 6A,B). The reduced sensitivity of autolysosomes in *ptl-1* mutant to BA1 suggests that fusion events between autophagosomes and lysosomes might be reduced in *ptl-1* mutants.

## 3. Discussion

Accumulation of misfolded proteins is a common pathology shared by various neurodegenerative diseases. Since autophagy is a conserved mechanism that maintain cellular homeostasis by degrading damaged organelles and misfolded proteins, autophagy activity can affect the onset and progression of neurodegenerative diseases, with which Autophagy dysfunction is often associated. However, it remains unclear whether autophagy impairment is a contributor or consequence of neurodegeneration. In this study, we examined the autophagic response to axon injury in a *C. elegans* model of tauopathy that expresses pro-aggregant Tau fragment. We showed that injury-triggered autophagy induction is impaired in neurons with Tau aggregation, demonstrating the detrimental effect of Tau aggregation on autophagy regulation.

This impaired neuronal autophagy response to axon injury is also found in aged neurons [30]. Numerous studies have reported that, as the organism ages, regulation of protein homeostasis becomes disrupted, resulting in accumulation of misfolded proteins. In *C. elegans*, a sharp decline in chaperone expression correlates with the end of the reproductive phase and leads to the aggregation of misfoled mutant proteins [49]. In mammals, the ER stress-induced unfolded protein response is impaired with age [50]. Similarly, lysosomal chaperone-mediated autophagy activity is reduced in old-aged rat livers and senescent human fibroblasts [51]. It’s also known that physiological age-related aggregates resemble disease aggregates in several aspects [52]. Therefore, the impaired autophagic response in aged neuron could be partially due to age-related protein aggregation. However, our data on rapamycin treatment suggest that Tau aggregation and aging might impact autophagy through different mechanisms. We have previously shown that treating young adult animals with autophagy-inducing agents leads to elevated autophagosome and autolysosome numbers in un-injured neurons. However, in injured young neurons, which have high level of autophagy activity in response to injury, autophagy-activating drugs do not further activate autophagy. Additionally, these autophagy activating agents were sufficient to increase autophagic vesicle number in aged wild-type neurons. These summarized observations suggest that axonal injury maximally activates autophagy in young wild-type neurons. This may explain the failure of autophagy-inducing agents to further augment autophagy. However, in neurons with defective injury response, such as those in aged animals, autophagy activity is not at the maximal level and therefore can be elevated by autophagy-activating agents. Interestingly, in neurons expressing pro-aggregant Tau, rapamycin treatment was not sufficient to enhance the number of autophagy vesicles, despite the low level in these neurons.

Tau aggregation is often associated with Tau hyperphosphorylation and loss of Tau-MT interaction in Tau-related neurodegeneration [42]. The involvement of MTs in different steps of autophagic process has been a debatable topic over the past years, but it becomes clear that MTs play essential roles in regulating autophagy dynamics. In this study, we found that loss of PTL-1/Tau lead to enhanced basal level of autophagy, inconsistent with previous reports that acute treatment of nocodazole or Taxol did not affect basal autophagosome formation [45,46,47]. We suspect that the high basal autophagy level of autophagy in *ptl-1* mutant might be due to a stress-like condition caused by the loss of PTL-1 function in MT assembly and stabilization. Although it is generally believed that basal autophagosome formation does not involve MT, stress-induced autophagosome formation requires proper MT function [48]. WIPI1-positive pre-autophagosomal structures move along MTs upon starvation of the cells and such movements are highly sensitive to nocodazole treatment [53]. MTs and MT motors are also known to regulate mTORC1 and the class III PI3-kinase complex, the two major complexes required to initiate the autophagic response [54]. We found that either axotomy stress or rapamycin was sufficient to promote autophagic vesicle formation in *ptl-1* mutant (Figure 5), indicating the important role of MTs in neuronal autophagy regulation in response to axon injury. Furthermore, the high level of autolysosomes in *ptl-1* mutant is relatively more resistant to BA1, which inhibits the fusion of autophagosomes with lysosomes. This suggests that fusion events might be reduced in *ptl-1* mutants, despite the high basal autophagy level. This is consistent with the important role of MTs in regulating the movement of autophagosome toward the perinuclear region, where autophagosomes fuse with lysosomes [55]. We showed that injury-triggered autophagy induction is negatively impacted by both Tau aggregation and PTL-1/Tau deletion. However, elevated basal autophagy level is only observed in *ptl-1* mutant, but not in Tau transgenic animals, suggesting that Tau aggregation and PTL-1/Tau loss-of-function might affect autophagy regulation through distinct mechanisms. Extensive studies have demonstrated that Alzheimer’s disease is associated with defects in different steps of autophagy, including initiation, autophagosome transport and lysosomal fusion [56]. These defects are likely due to a combined effect of protein aggregation and abnormal MT function.

In summary, we employed a dual reporter to monitor the dynamics of autophagosomes and autolysosomes in response to axon injury. We found that this injury-induced autophagy response was abolished in neurons expressing pro-aggregant Tau and neurons with PTL-1/Tau depletion, and that rapamycin was not sufficient to restore these defects in autophagy response, suggesting that aberrant protein aggregation or abnormal MT function can modulate autophagy regulation in neurons after injury through distinct mechanisms.

## 4. Materials and Methods

### 4.1. C. elegans Genetics

*C. elegans* strains were maintained at 20 °C using standard procedures on nematode growth medium (NGM) agar plates with OP50 *E. coli*. Mutant *C. elegans* strains carrying *ptl-1(ok621)* alleles and transgenic Tau strains carrying *byIs161*, and *byIs162* alleles were provided by the *C. elegans* gene knockout consortium (CGC). This consortium is funded by the NIH Office of Research Infrastructure Programs (P40 OD010440). Microinjection was utilized to generate transgenic animals. PCR was used for genotyping.

### 4.2. Transgene Construction

To construct the P*mec-4::mCherry::gfp::lgg-1* vector, *mCherry::gfp::lgg-1* sequence was amplified by PCR from the lysate of MAH215 (Chang et al., 2017) and PCR product was inserted into TOPO TA vector to generate entry vectors. The expression vectors were generated using LR gateway recombination between the entry vectors and the P*mec-4* gateway cloning destination vector.

### 4.3. Laser Axotomy

Axotomy was performed as previously described by Wu et al. [57], with slight modification. Batches of 12 adult day 1 animals, unless otherwise indicated, were first immobilized using either 0.7% phenoxypropanol (484423, MilliporeSigma, St. Louis, MO, USA) or muscimol (M1523, MilliporeSigma, St. Louis, MO, USA). An Olympus IX83 microscope using 100× objective was then used to visualize GFP labeled PLM axons. Axotomies were performed with a Micropoint UV laser (Andor Technology, Oxford Instrument, Belfast, United Kingdom) at 50 µm from the cell body. Animals were then recovered in agar plates for 24 h prior to remounting, unless otherwise indicated, for scoring. A minimum of 20 animals were axotimized. ImageJ was used to measure 10 axons of each genotype. All experiments performed with a matched control in parallel.

### 4.4. Rapamycin and Bafilomycin A1 treatment

Rapamycin (AG-CN2-0025, AdipoGen, San Diego, CA, USA) was first dissolved in DMSO at a final concentration of 100 nM. This mixture was then added to standard NGM agar plates. Administration of rapamycin was performed by culturing on rapamycin NGM plates for 24 h prior to imaging, unless otherwise indicated. Bafilomycin A1 (BA1; BBVT-0252-C100, BioViotica, Dransfeld, Germany) was suspended in 0.2% DMSO and 0.1% phenol red solution at a final concentration of 50 µM and administered via injection into body cavity at tail position. This was done as BA1 is not cuticle permeable. Injections were performed 24 h before imaging. For axotomy experiments involving BA1 treatment, animals were cultured on NGM plates for 2 h post injection prior to axotomy.

### 4.5. Quantification of Autophagic Vesicles

Live animals were first immobilized with either 0.7% phenoxypropanol or 30 mM muscimol on a 5% agarose pad for quantitative analysis of both vesicles and puncta. Fluorescence images were taken on either an Olympus IX83 fluorescence microscope or a Zeiss LSM780 confocal microscope at 100× or 63× oil objectives. Confocal Z-stack images were generated with 0.5 µm slice intervals. Autophagic vesicle puncta in PLM neuron cell bodies were manually counted. Puncta were labeled by GFP::LGG-1 and mCherry::LGG-1. In neurons with the tandem sensor, total puncta were counted of either only mCherry-positive, only GFP-positive, or mCherry and GFP double-positive. Autolysosome puncta, indicated by mCherry-only, were counted subsequent to merging separate red and green channel images. Autolysosome puncta was verified by substracting the total number of GFP-positive puncta from the total number of mCherry-positive puncta.

### 4.6. Live imaging of Autophagy Dynamics

Live imaging of autophagic vesicles was performed 24 h after axotomy. Videos were taken with an Olympus IX83 microscope. Each video was 5 min in duration. Kymographs were generated with the software Kymograph of the Olympus IX83. The kymographs were utilized to determine autophagic vesicle trafficking. Kymographs are two-dimensional displays of a video with the *x*-axis is a line-scan of each video frame at a particular time, representing space along the process, and the *y*-axis representing time. This sequential assembly visualizes autophagic vesicle behavior over time.

### 4.7. Statistical Analysis

The software GraphPad PRiSM (version 8, San Diego, CA, USA) was utilized for all statistical analysis. Student’s t tests were utilized for all two-way comparisons. ANOVA tests were performed for comparisons involving multiple groups in the design.

## Abbreviations

AL	Autolysosome
AP	Autophagosome
BA1	Bafilomycin A1
CNS	Central Nervous System
LGG-1	LC3, GABARAP and GATE-16 family-1
MT	Microtubule
PLM	Posterior Lateral Mechanosensory
PNS	Peripheral Nervous System
PTL-1	Protein with Tau-like repeats-1

## References

[1] Kroemer G., Marino G., Levine B. (2010). Autophagy and the integrated stress response. Mol. Cell.

[2] Levine B., Klionsky D.J. (2004). Development by self-digestion: Molecular mechanisms and biological functions of autophagy. Dev. Cell.

[3] Mizushima N., Levine B., Cuervo A.M., Klionsky D.J. (2008). Autophagy fights disease through cellular self-digestion. Nature.

[4] Ravanan P., Srikumar I.F., Talwar P. (2017). Autophagy: The spotlight for cellular stress responses. Life Sci..

[5] Mizushima N., Yoshimori T., Levine B. (2010). Methods in mammalian autophagy research. Cell.

[6] Metaxakis A., Ploumi C., Tavernarakis N. (2018). Autophagy in Age-Associated Neurodegeneration. Cells.

[7] Komatsu M., Waguri S., Chiba T., Murata S., Iwata J., Tanida I., Ueno T., Koike M., Uchiyama Y., Kominami E. (2006). Loss of autophagy in the central nervous system causes neurodegeneration in mice. Nature.

[8] Berger Z., Ravikumar B., Menzies F.M., Oroz L.G., Underwood B.R., Pangalos M.N., Schmitt I., Wullner U., Evert B.O., O’Kane C.J. (2006). Rapamycin alleviates toxicity of different aggregate-prone proteins. Hum. Mol. Genet..

[9] Ravikumar B., Vacher C., Berger Z., Davies J.E., Luo S., Oroz L.G., Scaravilli F., Easton D.F., Duden R., O’Kane C.J. (2004). Inhibition of mTOR induces autophagy and reduces toxicity of polyglutamine expansions in fly and mouse models of Huntington disease. Nat. Genet..

[10] Mandrioli J., D’Amico R., Zucchi E., Gessani A., Fini N., Fasano A., Caponnetto C., Chio A., Dalla Bella E., Lunetta C. (2018). Rapamycin treatment for amyotrophic lateral sclerosis: Protocol for a phase II randomized, double-blind, placebo-controlled, multicenter, clinical trial (RAP-ALS trial). Medicine.

[11] Schaeffer V., Lavenir I., Ozcelik S., Tolnay M., Winkler D.T., Goedert M. (2012). Stimulation of autophagy reduces neurodegeneration in a mouse model of human tauopathy. Brain.

[12] Lin A.L., Jahrling J.B., Zhang W., DeRosa N., Bakshi V., Romero P., Galvan V., Richardson A. (2017). Rapamycin rescues vascular, metabolic and learning deficits in apolipoprotein E4 transgenic mice with pre-symptomatic Alzheimer’s disease. J. Cereb. Blood Flow Metab..

[13] Ozcelik S., Fraser G., Castets P., Schaeffer V., Skachokova Z., Breu K., Clavaguera F., Sinnreich M., Kappos L., Goedert M. (2013). Rapamycin attenuates the progression of tau pathology in P301S tau transgenic mice. PLoS ONE.

[14] Siman R., Cocca R., Dong Y. (2015). The mTOR Inhibitor Rapamycin Mitigates Perforant Pathway Neurodegeneration and Synapse Loss in a Mouse Model of Early-Stage Alzheimer-Type Tauopathy. PLoS ONE.

[15] Congdon E.E., Wu J.W., Myeku N., Figueroa Y.H., Herman M., Marinec P.S., Gestwicki J.E., Dickey C.A., Yu W.H., Duff K.E. (2012). Methylthioninium chloride (methylene blue) induces autophagy and attenuates tauopathy in vitro and in vivo. Autophagy.

[16] Kim S., Choi K.J., Cho S.J., Yun S.M., Jeon J.P., Koh Y.H., Song J., Johnson G.V., Jo C. (2016). Fisetin stimulates autophagic degradation of phosphorylated tau via the activation of TFEB and Nrf2 transcription factors. Sci. Rep..

[17] Kruger U., Wang Y., Kumar S., Mandelkow E.M. (2012). Autophagic degradation of tau in primary neurons and its enhancement by trehalose. Neurobiol. Aging.

[18] Wang Y., Mandelkow E. (2012). Degradation of tau protein by autophagy and proteasomal pathways. Biochem. Soc. Trans..

[19] Lim F., Hernandez F., Lucas J.J., Gomez-Ramos P., Moran M.A., Avila J. (2001). FTDP-17 mutations in tau transgenic mice provoke lysosomal abnormalities and Tau filaments in forebrain. Mol. Cell. Neurosci..

[20] Wang Y., Song M., Song F. (2018). Neuronal autophagy and axon degeneration. Cell Mol. Life Sci..

[21] Komatsu M., Wang Q.J., Holstein G.R., Friedrich V.L., Iwata J., Kominami E., Chait B.T., Tanaka K., Yue Z. (2007). Essential role for autophagy protein Atg7 in the maintenance of axonal homeostasis and the prevention of axonal degeneration. Proc. Natl. Acad. Sci. USA.

[22] Maday S. (2016). Mechanisms of neuronal homeostasis: Autophagy in the axon. Brain Res..

[23] Nishiyama J., Miura E., Mizushima N., Watanabe M., Yuzaki M. (2007). Aberrant membranes and double-membrane structures accumulate in the axons of Atg5-null Purkinje cells before neuronal death. Autophagy.

[24] He Z., Jin Y. (2016). Intrinsic Control of Axon Regeneration. Neuron.

[25] Kanno H., Ozawa H., Sekiguchi A., Itoi E. (2009). The role of autophagy in spinal cord injury. Autophagy.

[26] Rodriguez-Muela N., Boya P. (2012). Axonal damage, autophagy and neuronal survival. Autophagy.

[27] Lipinski M.M., Wu J., Faden A.I., Sarkar C. (2015). Function and Mechanisms of Autophagy in Brain and Spinal Cord Trauma. Antioxid. Redox Sign..

[28] He M., Ding Y., Chu C., Tang J., Xiao Q., Luo Z.G. (2016). Autophagy induction stabilizes microtubules and promotes axon regeneration after spinal cord injury. Proc. Natl. Acad. Sci. USA.

[29] Saraswat Ohri S., Bankston A.N., Mullins S.A., Liu Y., Andres K.R., Beare J.E., Howard R.M., Burke D.A., Riegler A.S., Smith A.E. (2018). Blocking Autophagy in Oligodendrocytes Limits Functional Recovery after Spinal Cord Injury. J. Neurosci. Off. J. Soc. Neurosci..

[30] Ko S.H., Apple E.C., Liu Z., Chen L. (2020). Age-dependent autophagy induction after injury promotes axon regeneration by limiting NOTCH. Autophagy.

[31] Fatouros C., Pir G.J., Biernat J., Koushika S.P., Mandelkow E., Mandelkow E.M., Schmidt E., Baumeister R. (2012). Inhibition of tau aggregation in a novel Caenorhabditis elegans model of tauopathy mitigates proteotoxicity. Hum. Mol. Genet..

[32] Monaco A., Fraldi A. (2020). Protein Aggregation and Dysfunction of Autophagy-Lysosomal Pathway: A Vicious Cycle in Lysosomal Storage Diseases. Front. Mol. Neurosci..

[33] Wang Y.P., Biernat J., Pickhardt M., Mandelkow E., Mandelkow E.M. (2007). Stepwise proteolysis liberates tau fragments that nucleate the Alzheimer-like aggregation of full-length tau in a neuronal cell model. Proc. Natl. Acad. Sci. USA.

[34] Wang Y., Martinez-Vicente M., Kruger U., Kaushik S., Wong E., Mandelkow E.M., Cuervo A.M., Mandelkow E. (2009). Tau fragmentation, aggregation and clearance: The dual role of lysosomal processing. Hum. Mol. Genet..

[35] Kimura S., Noda T., Yoshimori T. (2007). Dissection of the autophagosome maturation process by a novel reporter protein, tandem fluorescent-tagged LC3. Autophagy.

[36] Chang J.T., Kumsta C., Hellman A.B., Adams L.M., Hansen M. (2017). Spatiotemporal regulation of autophagy during Caenorhabditis elegans aging. eLife.

[37] Hubert T., Wu Z., Chisholm A.D., Jin Y. (2014). S6 kinase inhibits intrinsic axon regeneration capacity via AMP kinase in Caenorhabditis elegans. J. Neurosci. Off. J. Soc. Neurosci..

[38] Ghosh-Roy A., Wu Z., Goncharov A., Jin Y., Chisholm A.D. (2010). Calcium and cyclic AMP promote axonal regeneration in Caenorhabditis elegans and require DLK-1 kinase. J. Neurosci. Off. J. Soc. Neurosci..

[39] Maday S., Wallace K.E., Holzbaur E.L. (2012). Autophagosomes initiate distally and mature during transport toward the cell soma in primary neurons. J. Cell Biol..

[40] Jahreiss L., Menzies F.M., Rubinsztein D.C. (2008). The itinerary of autophagosomes: From peripheral formation to kiss-and-run fusion with lysosomes. Traffic.

[41] Monastyrska I., Rieter E., Klionsky D.J., Reggiori F. (2009). Multiple roles of the cytoskeleton in autophagy. Biol. Rev. Camb. Philos. Soc..

[42] Barbier P., Zejneli O., Martinho M., Lasorsa A., Belle V., Smet-Nocca C., Tsvetkov P.O., Devred F., Landrieu I. (2019). Role of Tau as a Microtubule-Associated Protein: Structural and Functional Aspects. Front. Aging Neurosci..

[43] Goedert M., Baur C.P., Ahringer J., Jakes R., Hasegawa M., Spillantini M.G., Smith M.J., Hill F. (1996). PTL-1, a microtubule-associated protein with tau-like repeats from the nematode Caenorhabditis elegans. J. Cell Sci..

[44] McDermott J.B., Aamodt S., Aamodt E. (1996). ptl-1, a Caenorhabditis elegans gene whose products are homologous to the tau microtubule-associated proteins. Biochemistry.

[45] Aplin A., Jasionowski T., Tuttle D.L., Lenk S.E., Dunn W.A. (1992). Cytoskeletal elements are required for the formation and maturation of autophagic vacuoles. J. Cell. Physiol..

[46] Kochl R., Hu X.W., Chan E.Y., Tooze S.A. (2006). Microtubules facilitate autophagosome formation and fusion of autophagosomes with endosomes. Traffic.

[47] Reunanen H., Marttinen M., Hirsimaki P. (1988). Effects of griseofulvin and nocodazole on the accumulation of autophagic vacuoles in Ehrlich ascites tumor cells. Exp. Mol. Pathol..

[48] Mackeh R., Perdiz D., Lorin S., Codogno P., Pous C. (2013). Autophagy and microtubules—New story, old players. J. Cell Sci..

[49] Ben-Zvi A., Miller E.A., Morimoto R.I. (2009). Collapse of proteostasis represents an early molecular event in Caenorhabditis elegans aging. Proc. Natl. Acad. Sci. USA.

[50] Brown M.K., Naidoo N. (2012). The endoplasmic reticulum stress response in aging and age-related diseases. Front. Physiol..

[51] Cuervo A.M., Dice J.F. (2000). Age-related decline in chaperone-mediated autophagy. J. Biol. Chem..

[52] Hipp M.S., Kasturi P., Hartl F.U. (2019). The proteostasis network and its decline in ageing. Nat. Rev. Mol. Cell Biol..

[53] Geeraert C., Ratier A., Pfisterer S.G., Perdiz D., Cantaloube I., Rouault A., Pattingre S., Proikas-Cezanne T., Codogno P., Pous C. (2010). Starvation-induced hyperacetylation of tubulin is required for the stimulation of autophagy by nutrient deprivation. J. Biol. Chem..

[54] Sancak Y., Bar-Peled L., Zoncu R., Markhard A.L., Nada S., Sabatini D.M. (2010). Ragulator-Rag complex targets mTORC1 to the lysosomal surface and is necessary for its activation by amino acids. Cell.

[55] Kimura S., Noda T., Yoshimori T. (2008). Dynein-dependent movement of autophagosomes mediates efficient encounters with lysosomes. Cell Struct. Funct..

[56] Liu J., Li L. (2019). Targeting Autophagy for the Treatment of Alzheimer’s Disease: Challenges and Opportunities. Front. Mol. Neurosci..

[57] Wu Z., Ghosh-Roy A., Yanik M.F., Zhang J.Z., Jin Y., Chisholm A.D. (2007). Caenorhabditis elegans neuronal regeneration is influenced by life stage, ephrin signaling, and synaptic branching. Proc. Natl. Acad. Sci. USA.

